# Assessment of quality and reliability of Chinese-language myocardial infarction content on Bilibili and TikTok: a cross-sectional study

**DOI:** 10.3389/fpubh.2026.1748168

**Published:** 2026-03-19

**Authors:** Qingruo Mao, Li Zhong, Songzi Wang

**Affiliations:** 1 Department of Acupuncture, Xiyuan Hospital, China Academy of Chinese Medical Sciences, Beijing, China; 2 China Academy of Chinese Medical Sciences Data Center, Beijing, China

**Keywords:** Bilibili, health education, myocardial infarction, social media, TikTok, video quality

## Abstract

**Background:**

Myocardial infarction (MI) is a leading cause of cardiovascular mortality worldwide and requires timely treatment and accurate public awareness of risk factors, warning signs, and first aid. In China, short-video platforms such as TikTok (Douyin, Chinese mainland version) and Bilibili have become major health information sources, yet the quality and reliability of MI-related content remain inadequately evaluated.

**Objective:**

This cross-sectional study systematically assessed and compared the quality, reliability, and educational value of MI-related videos on TikTok and Bilibili.

**Methods:**

Using the keyword “心肌梗死” (myocardial infarction), we retrieved the top 100 videos from TikTok and Bilibili on September 1, 2025. After exclusions, 137 videos were included. Uploaders were classified as clinicians, patients, or traditional Chinese medicine practitioners. Quality was evaluated using GQS, mDISCERN, JAMA benchmarks, and PEMAT-U/A. Statistical analyses included Spearman correlation, Mann–Whitney *U*, Kruskal–Wallis, and chi-square tests.

**Results:**

Bilibili videos were significantly longer but had much lower engagement than TikTok videos. Only mDISCERN scores differed significantly between platforms: Bilibili contained a higher proportion of high-reliability videos than TikTok, while JAMA, GQS, and PEMAT-U/A scores did not differ significantly. Uploader background significantly influenced quality outcomes. Clinicians and TCM practitioners achieved higher JAMA scores than patients, indicating greater formal credibility, whereas patients had a higher proportion of high mDISCERN scores, reflecting more detailed experiential content. Correlation analysis revealed a bidirectional effect of video length: longer duration was positively associated with mDISCERN and GQS scores but negatively associated with JAMA scores. Interaction metrics showed strong internal synergy but almost no correlation with professional quality scores, demonstrating a clear “quality popularity paradox.”Content analysis showed an imbalanced pattern: particularly regarding emergency measures and medication safety information were severely lacking.

**Conclusion:**

MI-related content on Chinese short-video platforms is of moderate quality but characterized by a significant disconnect between popularity and educational value, as well as critical deficiencies in emergency response information. These findings underscore the urgent need for coordinated interventions, including platform-level quality control, collaborative content creation between professionals and platform, and enhanced public health literacy to ensure the safe and effective use of these platforms for health education.

## Introduction

Myocardial infarction (MI) is a cardiovascular disease characterized by myocardial necrosis, caused by acute and persistent ischemia and hypoxia of the coronary arteries. Notably, it is among the leading causes of death among patients with cardiovascular diseases (1). Globally, the MI prevalence is becoming increasingly high, causing > 15% mortality each year and posing a major threat to human health (2, 3). The INTERHEART study, covering 52 countries worldwide, demonstrated that 9 risk factors explained 90 and 94% of the risk of MI in men and women, respectively, in all regions, sexes, and age groups (4). This includes smoking, dyslipidemia, hypertension, diabetes, abdominal obesity, psychosocial factors, insufficient fruit and vegetable intake, inappropriate alcohol consumption, and lack of regular physical exercise. Implementing lifestyle interventions for high-risk individuals, such as smoking cessation, dietary adjustment, and increased physical activity, can significantly reduce MI incidence (5). Therefore, educational measures and lifestyle behavior changes are core strategies for preventing MI, which need to be promoted through public health policies and individual actions.

In the digital era, electronic information has replaced paper-based information as the primary means for the public to access information. Among Chinese various platforms, short video platforms, such as TikTok and Bilibili, have gradually become important sources of health information because of their intuitiveness, high transmission efficiency, and wide coverage (6). The core advantages of these platforms include the following: (1) information presentation intuitiveness: use of short videos to dynamically demonstrate the warning signs and prevention measures of MI, thereby improving the understanding efficiency of patients; (2) multi-source information complementarity: different content creators, such as doctors, patients, and institutions, provide diverse perspectives, helping patients verify the information obtained from hospitals and reducing the distrust caused by information asymmetry between doctors and patients; (3) time and space accessibility: overcoming the time and location constraints of medical consultations, reducing transportation costs and infection risks, and improving the treatment compliance of patients; and (4) precise recommendation mechanisms: algorithm-based recommendation systems can target specific patient groups, enhancing the efficiency of information dissemination (6, 7). Nonetheless, the following shortcomings cannot be ignored: (1) lack of quality control: among the massive amount of user-generated content, low-quality or misleading information spread by non-professionals may endanger public health, such as exaggerating surgical effects or promoting non-standard treatments; and (2) interference from commercial interests: some doctors release biased content for self-promotion, which undermines information reliability (8). Currently, although short video platforms have become a “key repository” of public disease information, systematic quality assessment of MI-related videos, including credibility, educational value, and influencing factors, remains lacking. Similar concerns regarding variability in cardiovascular educational content have been reported internationally. For example, Tezcan and Akyildiz Tezcan evaluated cardiac rehabilitation videos on YouTube and found substantial heterogeneity in reliability and educational depth, as well as a weak correlation between popularity metrics and information quality (9). These findings underscore the need for platform-specific assessments across different cultural and linguistic contexts. Therefore, this study aimed to evaluate the quality of MI content on TikTok and Bilibili using established assessment systems such as the *Journal of the American Medical Association* (JAMA) benchmark criteria, global quality scale (GQS), and modified DISCERN (mDISCERN), to examine how video duration and source and content type affect video quality and propose regulatory and professional collaboration strategies, including establishing content review standards and encouraging medical teams to create content. Through this research, we aimed to provide empirical evidence for digital health communication and emphasizes the necessity of strengthening platform regulation and professional participation.

## Methods

### Study design

This was a cross-sectional observational study of publicly available short videos related to myocardial infarction (MI) on two major Chinese video-sharing platforms TikTok and Bilibili. The study aimed to assess the quality, reliability, and educational value of MI-related content at a single time point, without intervention or longitudinal follow-up.

### Video collection methods

Content at a single time point, without intervention or longitudinal follow-up.

Data collection was conducted entirely online in a logged-out browser environment to minimize the influence of personalized algorithms and prior browsing history. All videos analyzed were publicly accessible and did not contain identifiable personal information.

### Video collection time

The data capture date was September 1, 2025. Due to dynamic ranking updates by social media algorithms, all searches were completed within a 3-h window on that day. Before each platform search, users logged out, cleared browser cache and cookies, and disabled location-based VPN operations. All video engagement metrics (likes, comments, saves, shares, and watch time) were extracted simultaneously to minimize temporal bias from platform interaction dynamics. Videos released after this date were excluded from the statistical analysis.

### Data sources and video selection

We searched from TikTok (Douyin, Chinese mainland version) and Bilibili, two major Chinese video-sharing platforms using the Chinese keyword “心肌梗死” (myocardial infarction). The top 100 videos ranked by each platform’s “comprehensive recommendation” algorithm were initially retrieved.

### Exclusion criteria

**Figure 1:**
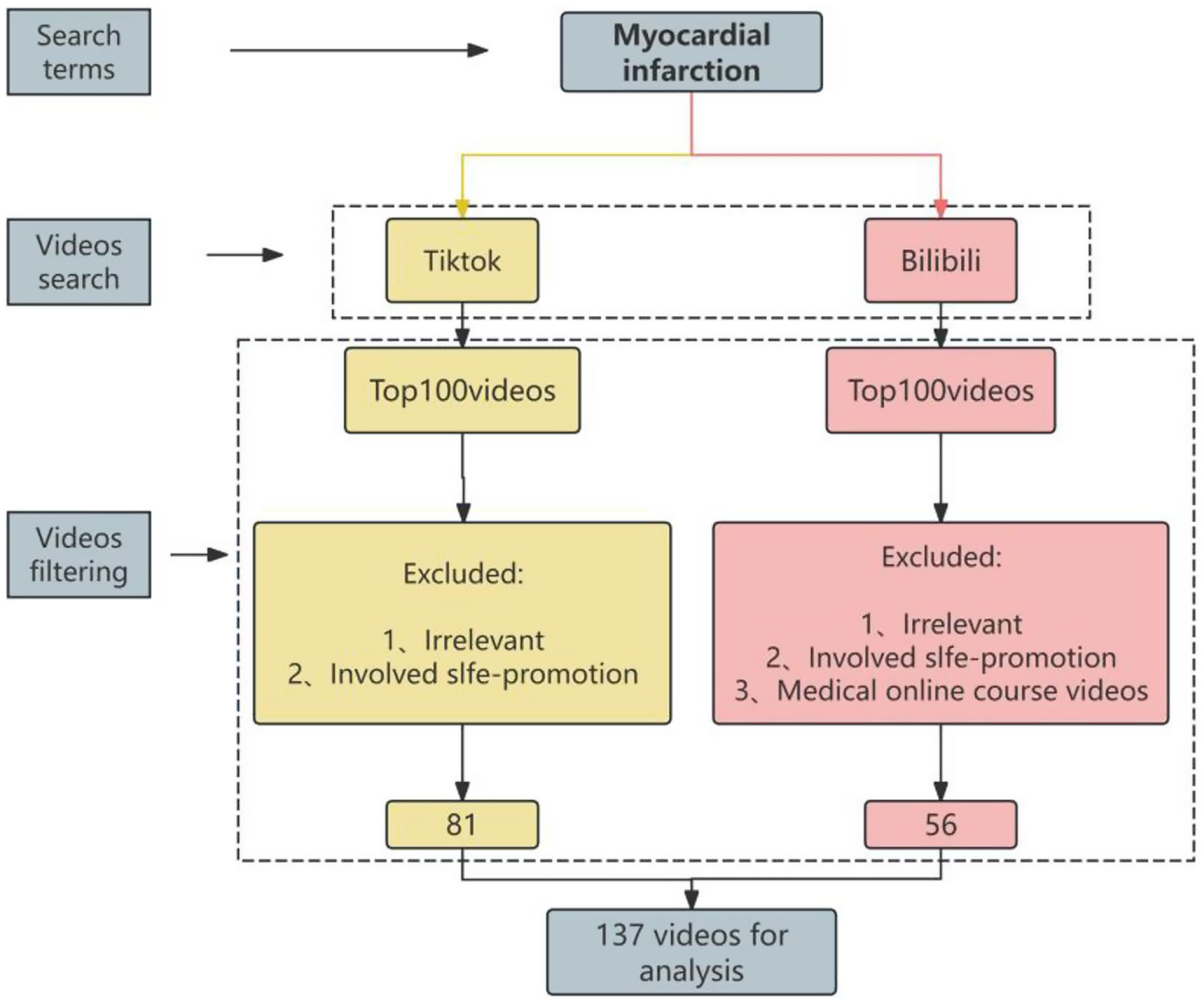
Flowchart of the video selection process for analyzing myocardial infarction content on TikTok and Bilibili.

Videos were excluded if they met any of the following conditions:

(1) Not directly related to MI treatment or management (e.g., focusing solely on pathology or diagnostics). (2) Promotional content for hospitals, clinics, or individual physicians. (3) Academic lectures or professional teaching materials aimed at medical students. After screening, 137 videos were eligible for analysis. The selection process is illustrated in Figure 1.

### Variables and data extraction

For each included video, the following variables were recorded in a standardized Excel spreadsheet: (1) Platform (TikTok or Bilibili), (2) Uploader identity, (3) Video duration (seconds), (4) Likes, (5) comments, (6) saves, (7) shares (engagement metrics).

(8) Classification of uploaders. Uploaders were categorized based on publicly visible profile information as: (1) Clinicians (licensed Western medical doctors), (2) Traditional Chinese Medicine (TCM) practitioners, (3) Patients/lay users. If classification was ambiguous, two independent reviewers discussed until consensus was reached, with arbitration by a senior physician when necessary.

### Quality and reliability assessment

Video quality and reliability were evaluated using four validated instruments:

(1) Global Quality Scale (GQS)—overall educational quality (1–5). (2) Modified DISCERN (mDISCERN)—reliability of health information (5 items). (3) JAMA Benchmark Criteria—credibility based on authorship, sourcing, timeliness, and conflicts of interest (0–4). (4) Patient Education Materials Assessment Tool (PEMAT-U/A)—understandability and actionability (0–100%) (10).

All reviewers underwent standardized training before scoring. Inter-rater discrepancies were resolved through discussion or senior adjudication.

### Content analysis

Video content was coded according to clinical MI management guidelines, focusing on whether videos addressed: (1) Lifestyle risk factors, (2) First-aid measures (e.g., chest compressions, AED use), (3) Pharmacological advice (e.g., nitroglycerin, aspirin, Suxiao Jiuxin Wan).

### Statistical analysis

The Shapiro–Wilk test was employed to assess the normality of the variables on the data extracted from the videos. For data with a parametric distribution, descriptive statistics were reported as mean and standard deviation. Non-normally distributed data were summarized using the median and interquartile range.

The Mann–Whitney *U* test was used for non-parametric comparisons between two independent groups, whereas the Kruskal–Wallis *H* test was implemented for comparisons among three or more groups. Spearman’s rank correlation coefficient (*r*) was used to evaluate the relationship between general information (likes, favorites, shares, comments, and video length), quality, and reliability scores. The intraclass correlation coefficient (ICC) was calculated using a two-way random-effects model to evaluate the consistency of scoring among reviewers.

Correlation strength was classified as follows: *r* < 0.2 (negligible), 0.2–0.4 (weak), 0.4–0.6 (moderate), 0.6–0.8 (strong), and > 0.8 (very strong). The chi-square test of independence was used to analyze associations between two variables. *p*-values < 0.05 were considered statistically significant. All statistical analyses were performed using R 4.4.0.

## Results

### Sample composition and overall quality level

**Figure 2:**
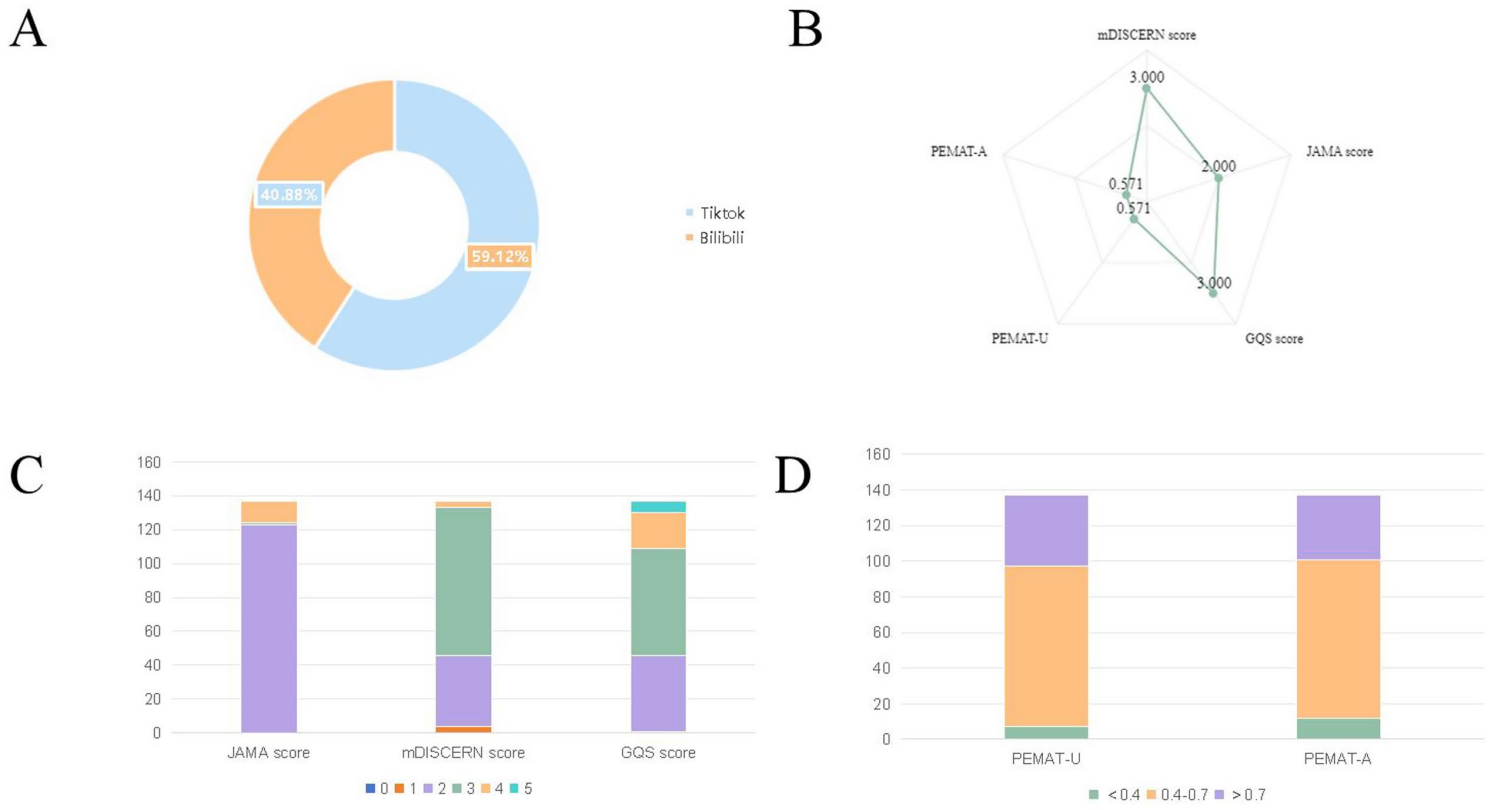
Distribution of overall conditions: **(A)** Proportion of myocardial infarction-related videos on TikTok and Bilibili; **(B)** radar chart of the JAMA, mDISCERN, GQS, and PEMAT-U/A scores; **(C)** proportion of videos with different JAMA, mDISCERN, and GQS scores; and **(D)** proportion of videos with different PEMAT-U/A scores. GQS, Global Quality Scale; JAMA, *Journal of the American Medical Association*; mDISCERN, modified DISCERN; PEMAT-A, Patient Education Materials Assessment Tool for Audiovisual materials; PEMAT-U, Patient Education Materials Assessment Tool for printable materials.

After applying the exclusion criteria, 137 videos were included in the analysis, with significant differences in the composition of the videos based on the platform: TikTok and Bilibili accounted for 59.12 and 40.88% of videos, respectively, indicating the dominance of the former (Figure 2A).

To evaluate the consistency of scoring among reviewers, inter-rater reliability was assessed prior to formal data analysis. Two independent reviewers underwent standardized training and independently evaluated a pilot set of 20 randomly selected videos using the four assessment instruments: GQS, mDISCERN, JAMA benchmarks, and PEMAT-U/A. The ICC values for the four tools ranged from 0.80 to 0.91, Indicates good inter-rater reliability and supports consistency in subsequent formal assessments.

The quality across dimensions generally exhibited a “concentration at the medium level, scarcity at the high level” pattern (Figure 2B), with the following specific distributions.

Regarding the JAMA score (for credibility), videos scoring 2 points were the most common (89.78%), with those scoring 3 and 4 points accounting for 0.73 and 9.49% of the videos, respectively. None of the videos had JAMA scores of 0, 1, and 5. The mean JAMA score was approximately 2.20, indicating that most videos reached a basic credibility level, yet content with higher credibility (3 or 4 points) was relatively scarce.

Regarding the mDISCERN score (for content reliability), videos scoring 3 points were most common (63.50%), followed by those scoring 2 points (30.66%), and those scoring 1 and 4 points each (2.92%). None of the videos had scores of 0 and 5. The mean mDISCERN score was approximately 2.66, indicating an overall reliability at a medium-to-high level, although content with the highest reliability (4 points) was rare.

The GQS score (overall quality) demonstrated a “high in the middle, low at both ends” distribution. Videos scoring 3 and 2 points accounted for 45.99 and 32.85%, respectively, whereas high-quality content (with scores of 4 and 5) was less than 21% (15.33 and 5.11%, respectively). A GQS score of 1 point was observed in only 0.73% of the videos. The mean GQS score was 2.912, indicating that the overall quality concentrated in the medium range.

The PEMAT-U score (for understandability) and PEMAT-A score (for actionability) showed a relatively balanced performance, mainly distributed in the 0.4–0.7 range, with some videos exceeding 0.7. The mean PEMAT-U and PEMAT-A scores were 55.89 ± 15.21 and 54.62 ± 15.87, respectively, suggesting that most videos exhibited medium-level information transmission efficiency and practical guidance value, with some demonstrating strong educational utility (Figures 2C,D).

### Differences between platforms

#### Video feature differences

##### Differences in video characteristics

Highly significant differences were observed between the two platforms in both video duration and interaction volume (*p* < 0.001).

Regarding video duration, Bilibili videos demonstrated a significantly higher median duration (82.00 s, P25 = 41.3 s, P75 = 102.8 s), with some exceeding 10 min, compared with TikTok videos, which were shorter (41.00 s, P25 = 18.0 s, P75 = 68.5 s), with > 60% of the videos having a duration within 1 min.

Regarding interaction volume, the median number of likes for TikTok videos (4852.00) was 30.3 times that for Bilibili videos (160.00), whereas the median number of comments (163.00) on TikTok videos was 32.6 times that on Bilibili videos (5.00). Significant differences between the platforms (TikTok VS Bilibili) were also observed in the number of favorites (1728.00 vs. 92.50) and shares (1014.00 vs. 44.00). Furthermore, high-interaction videos with over 100,000 likes accounted for 4.94% of TikTok videos, whereas Bilibili had no such videos.

##### Quality and reliability differences

**Figure 3:**
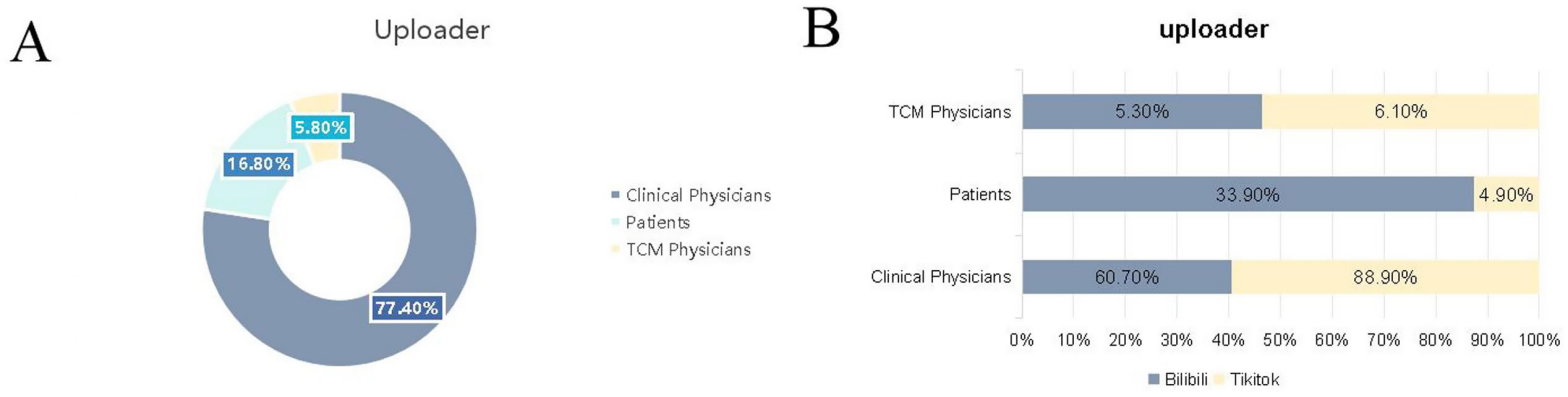
Proportion of videos stratified by uploader type **(A)** in the overall sample and **(B)** on Bilibili and TikTok platforms. TCM, traditional Chinese medicine.

Significant differences between the platforms were observed only in the mDISCERN score (reliability) (Mann–Whitney *U* = 1908.000, *z* = 3.003, *p* = 0.003), whereas the median score was 2.00 for both platforms. Overall, 17.86% of Bilibili videos scored 4 points, which was significantly higher than the corresponding proportion of 3.70% among TikTok videos (Figure 3).

**Figure 4:**
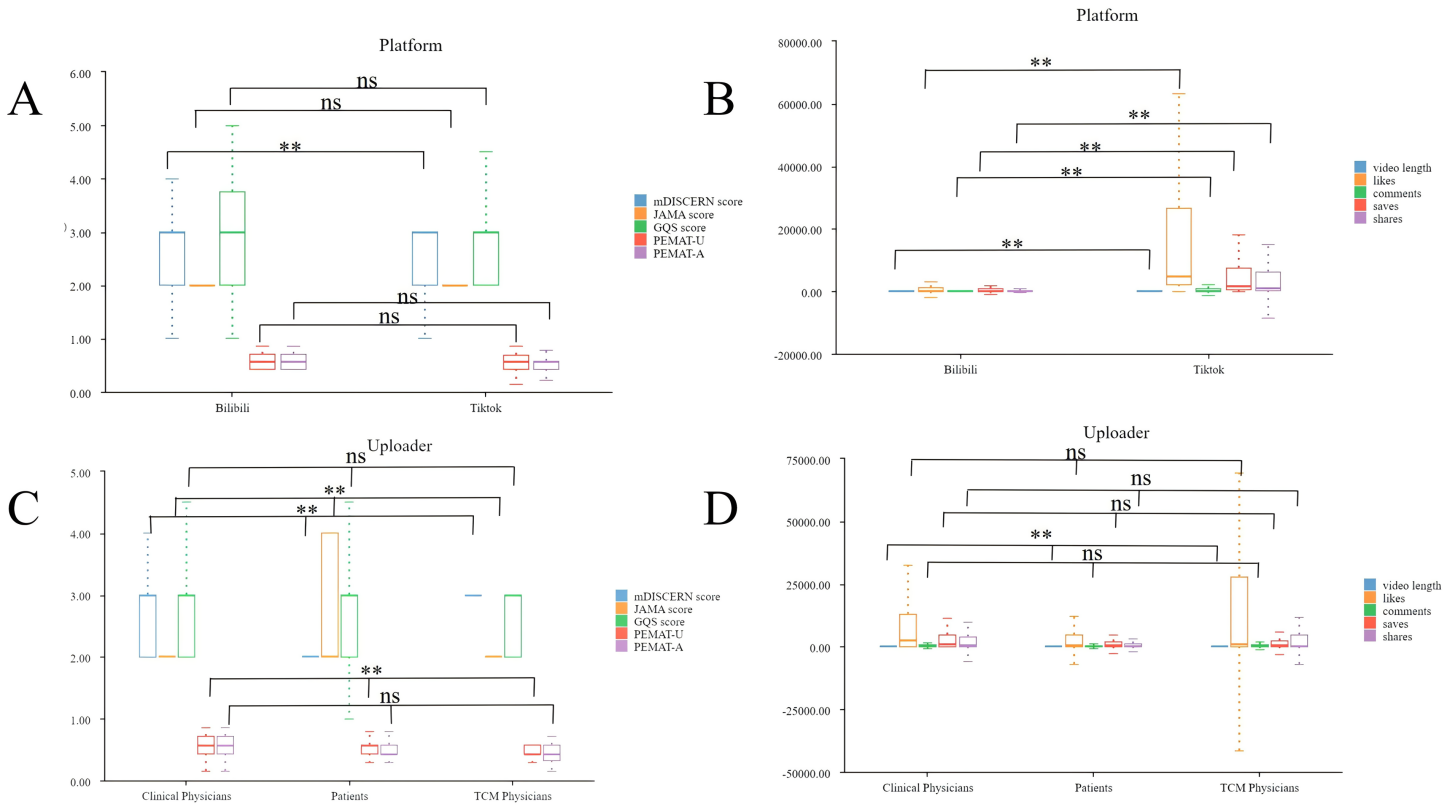
Box plots of platform- and uploader-related metrics. **(A)** Box plots of the mDISCERN, JAMA, GQS PEMAT-U, and PEMAT-A scores across platforms. **(B)** Box plots of video length, likes, comments, saves, and shares on myocardial infarction-related videos across platforms. **(C)** Box plots of the mDISCERN, JAMA, GQS PEMAT-U, and PEMAT-A scores among different types of uploaders. **(D)** Box plots of video length, likes, comments, saves, and shares among videos uploaded by different types of uploaders. “ns” indicates no statistically significant difference between groups; “**” indicates a significant difference between groups. GQS, global quality scale; JAMA, *Journal of the American Medical Association*; mDISCERN, modified DISCERN; PEMAT-A, patient education materials assessment tool for audiovisual materials; PEMAT-U, Patient education materials assessment tool for printable materials; TCM, traditional Chinese medicine.

No significant differences between the platforms were found in the JAMA, GQS, PEMAT-U, and PEMAT-A scores (*p* > 0.05), indicating that the two platforms performed comparably regarding basic credibility, overall quality, information understandability, and actionability (Table 1 and Figures 4A,B).

**Table 1:** Quality and reliability scores and overall information of videos on TikTok and Bilibili.

	Total (*n* = 137)	TikTok (*n* = 81)	Bilibili (*n* = 56)	*p*
Quality and reliability				
mDISCERN score M (Q1, Q3)	2.0 (2.0, 2.0)	2.0 (2.0, 2.0)	2.0 (2.0, 4.0)	0.009*
mDISCERN score mean ± SD	2.1 ± 0.5	2.0 ± 0.2	2.2 ± 0.7	0.009*
JAMA score M (Q1, Q3)	3.0 (2.0, 3.0)	3.0 (2.0, 3.0)	3.0 (2.0, 3.0)	0.014*
JAMA score mean ± SD	2.7 ± 0.6	2.7 ± 0.5	2.6 ± 0.7	0.014*
GQS score M (Q1, Q3)	3.0 (2.0, 3.0)	3.0(2.0, 3.0)	3.0(2.0, 3.0)	0.56
GQS score mean ± SD	3.0 ± 0.9	3.0 ± 0.8	3.0 ± 0.9	0.56
PEMAT-U score (%) M (Q1, Q3)	57.1 (42.9, 71.4)	57.1 (42.9, 71.4)	57.1 (42.9, 71.4)	0.343
PEMAT-U score (%) mean ± SD	55.9 ± 15.2	54.8 ± 16.0	57.5 ± 14.0	0.343
PEMAT-A score (%) M (Q1, Q3)	57.1 (42.9, 71.4)	57.1 (42.9, 71.4)	57.1 (42.9, 71.4)	0.114
PEMAT-A score (%) mean ± SD	54.6 ± 15.9	53.3 ± 16.6	56.7 ± 14.6	0.114
General information				
Video duration (s) M (Q1, Q3)	150.0 (66.0, 230.0)	122.0 (53.0, 188.0)	201.0 (84.0, 280.0)	0.036*
Video duration (s) mean ± SD	168.7 ± 245.4	135.5 ± 189.2	216.9 ± 301.6	0.036*
Likes M (Q1, Q3)	2800.0 (128.0, 13000.0)	7729.0 (2448.0, 26000.0)	304.0 (43.0, 3656.0)	0.001**
Likes mean ± SD	8965.4 ± 48215.7	11872.5 ± 55124.4	3842.6 ± 28754.2	0.001**
Comments M (Q1, Q3)	78.0 (8.0, 297.0)	236.0 (63.0, 556.0)	13.0 (3.0, 84.0)	0.001**
Saves M (Q1, Q3)	1492.0 (70.0, 4846.0)	4960.0 (926.0, 12000.0)	179.0 (18.0, 1479.0)	0.001**
Saves mean ± SD	3286.6 ± 15624.9	4325.7 ± 18752.4	987.4 ± 1056.8	0.001**

*p-*values are indicated (*p* < 0.05, **p < 0.001).

mDISCERN, modified DISCERN; JAMA, *Journal of the American Medical Association*, GQS, global quality scale; PEMAT-U, patient education materials assessment tool for understandability; PEMAT-A, patient education materials assessment tool for actionability; M, median; Q, quartile; SD, standard deviation.

#### Uploader attribute differences

##### Video feature differences

Highly significant differences in video duration were observed among different uploader groups (clinicians, patients, and TCM doctors). Patient-uploaded videos exhibited the longest median duration, at 93.00 s (P25 = 70.0 s, P75 = 111.0 s), followed videos uploaded by clinicians, at 46.50 s (P25 = 18.8 s, P75 = 79.0 s), and those uploaded by TCM doctors, at 29.50 s (P25 = 16.8 s, P75 = 50.0 s).

##### Interaction data differences

No significant differences in interaction volume (likes, comments, favorites, shares) were found among different uploader groups; nonetheless, certain patterns emerged. Regarding mean values, patient-uploaded videos received more likes (28568.39 ± 114631.07) than did those uploaded by clinicians and TCM doctors. When comparing median values, clinician-uploaded videos had more likes (2396.00) than did those uploaded by patients (609.00) and TCM doctors (1096.00); however, similar trends were observed for comments, favorites, and shares among the uploader groups.

##### Quality differences

Uploader attributes significantly influenced the JAMA, mDISCERN, and PEMAT-U scores.

Regarding the JAMA score (for credibility), videos uploaded by clinicians and TCM doctors scored significantly higher than those uploaded by patients; 17.39% of patient-uploaded videos had a JAMA score of 1, whereas this score was not observed among videos uploaded by clinicians and TCM doctors.

Regarding the mDISCERN score (for reliability), significant differences among the three uploader groups were found (*p* = 0.002); more patient-uploaded videos had a score of 4, compared with those uploaded by clinicians (30.43% vs. 5.66%). However, the median mDISCERN score was 2.00 for all uploader groups.

In terms of the PEMAT-U score (for understandability), TCM doctor-uploaded videos demonstrated the lowest median (0.429), significantly lower than that of videos uploaded by clinicians (0.571) and patients (0.571, Table 2 and Figures 4C,D).

**Table 2:** Quality and reliability scores and overall information of videos uploaded by different uploader groups.

	Total (*n* = 137)	Clinical physicians (*n* = 106)	Patients (*n* = 23)	TCM physicians (*n* = 8)	*p*
Quality and reliability					
mDISCERN score M(Q1, Q3)	2.0 (2.0, 2.0)	2.0 (2.0, 2.0)	2.0 (2.0, 4.0)	2.0 (2.0, 2.0)	0.006**
mDISCERN score mean ± SD	2.1 ± 0.5	2.5 ± 0.3	2.6 ± 0.9	2.0 ± 0.0	0.006**
JAMA score M (Q1, Q3)	3.0 (2.0, 3.0)	3.0 (2.0, 3.0)	2.0 (1.0, 2.0)	3.0 (2.0, 3.0)	0.000**
JAMA score mean ± SD	2.7 ± 0.6	2.7 ± 0.6	1.8 ± 0.6	2.9 ± 0.4	0.000**
GQS score M (Q1, Q3)	3.0 (2.0, 3.0)	3.0 (2.0, 3.0)	2.0 (2.0, 3.0)	3.0 (2.0, 3.0)	0.329
GQS score mean ± SD	3.0 ± 0.9	3.0 ± 0.9	2.7 ± 0.8	3.0 ± 0.9	0.329
PEMAT-U score M (Q1, Q3)	57.1 (42.9, 71.4)	57.1 (42.9, 71.4)	57.1 (42.9, 71.4)	42.9 (42.9, 57.1)	0.519
PEMAT-U score mean ± SD	55.9 ± 15.2	56.2 ± 15.0	54.4 ± 16.1	48.2 ± 10.7	0.519
PEMAT-A score M (Q1, Q3)	57.1 (42.9, 71.4)	57.1 (42.9, 71.4)	57.1 (42.9, 71.4)	37.5 (25.0, 50.0)	0.892
PEMAT-A score mean ± SD	54.6 ± 15.9	55.0 ± 15.7	53.9 ± 16.4	43.8 ± 15.3	0.892
General information					
Video duration M (Q1, Q3)	150.0 (66.0, 230.0)	156.0 (68.0, 240.0)	180.0 (90.0, 360.0)	60.0 (32.0, 132.0)	0.042*
Video duration mean ± SD	168.7 ± 245.4	172.4 ± 251.7	210.5 ± 312.8	85.5 ± 68.2	0.042*
Likes M (Q1, Q3)	2800.0 (128.0, 13000.0)	1280.0 (105.0, 7286.0)	28000.0 (1005.0, 553000.0)	48.0 (26.0, 443.0)	0.002**
Likes mean ± SD	8965.4 ± 48215.7	6542.8 ± 42156.4	28756.9 ± 125689.5	189.3 ± 176.8	0.002**
Comments M (Q1, Q3)	78.0 (8.0, 297.0)	58.0 (7.0, 297.0)	2356.0 (91.0, 12000.0)	1.0 (0.0, 8.0)	0.001**
Comments mean ± SD	386.4 ± 2015.7	298.6 ± 1875.4	2865.3 ± 8975.6	6.4 ± 7.2	0.001**
Saves M (Q1, Q3)	1492.0 (70.0, 4846.0)	847.0 (62.0, 4846.0)	15000.0 (243.0, 190000.0)	26.0 (7.0, 437.0)	0.001**
Saves mean ± SD	3286.6 ± 15624.9	2456.9 ± 14256.9	26897.5 ± 98756.3	156.8 ± 182.5	0.001**
Shares M (Q1, Q3)	633.0 (27.0, 1866.0)	463.0 (21.0, 1866.0)	4600.0 (178.0, 129000.0)	3.0 (1.0, 320.0)	0.001**
Shares mean ± SD	4892.6 ± 27568.9	3875.2 ± 25689.4	28965.4 ± 87654.2	98.6 ± 145.7	0.001**

*p*-values are indicated (p < 0.05, **p < 0.001).

TCM, traditional Chinese medicine; mDISCERN, modified DISCERN; JAMA, Journal of the American Medical Association, GQS, global quality scale; PEMAT-U, patient education materials assessment tool for understandability; PEMAT-A, patient education materials assessment tool for actionability; M, median; Q, quartile; SD, standard deviation.

### Influence of platform and uploader on high-score content distribution

**Figure 5:**
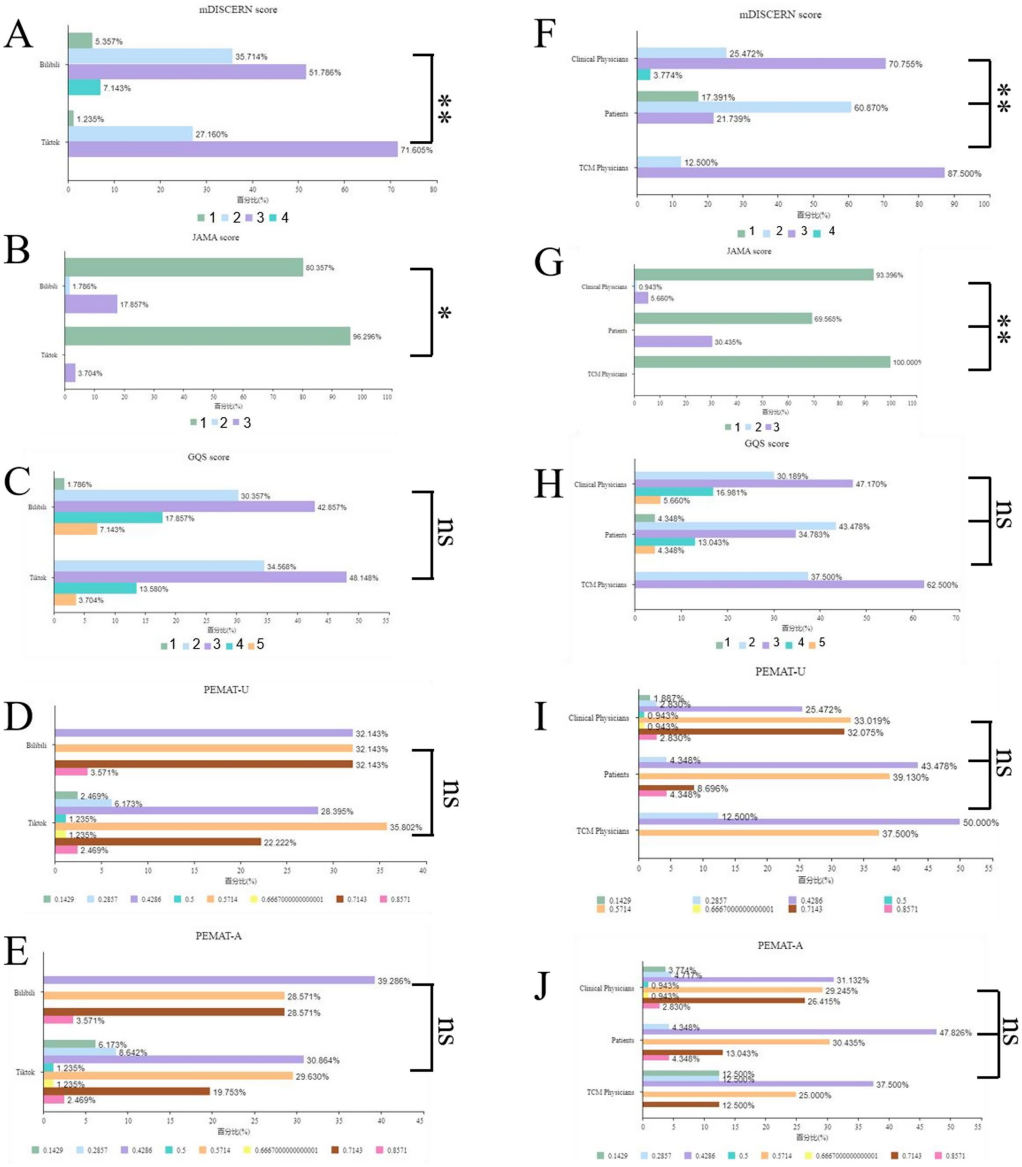
Distribution of chi-square test results for content scores stratified by platform and uploader type. **(A–E)** Chi-square test distributions of the mDISCERN, JAMA, GQS, PEMAT-U, and PEMAT-A scores across platforms. **(F–J)** Chi-square test distributions of the mDISCERN, JAMA, GQS, PEMAT-U, and PEMAT-A scores among different types of uploaders. GQS, Global Quality Scale; JAMA, *Journal of the American Medical Association*; mDISCERN, modified DISCERN; PEMAT-A, Patient Education Materials Assessment Tool for Audiovisual Materials; PEMAT-U, Patient Education Materials Assessment Tool for Printable Materials; TCM, traditional Chinese medicine.

Chi-square analysis demonstrated that both platform and uploader affected high-score content distribution without changing the “medium-quality dominance” pattern (Figure 5).

### Cross-association between platform and quality

Regarding the cross-association between the platform and JAMA score, Bilibili had 7.14% videos scoring 4 points, whereas TikTok had none (*χ*^2^ = 10.551, *p* = 0.014).

Regarding the cross-association between the platform and mDISCERN score, Bilibili and TikTok had 17.86 and 3.70% videos scoring 4 points, respectively (*χ*^2^ = 9.373, *p* = 0.009).

No significant differences were found in the cross-association analysis of platform with the GQS, PEMAT-U, and PEMAT-A scores (all *p* > 0.05), indicating that the overall quality of the videos on the two platforms was similar.

### Cross-association between uploader and quality

Regarding the cross-association between the uploader and JAMA score, 17.39% of patient-uploaded videos scored 1 point, whereas none of the videos uploaded by clinicians and TCM doctors showed this score (*χ*^2^ = 37.555, *p* < 0.001).

Regarding the cross-association between the uploader and mDISCERN score, 30.43 and 5.66% of videos uploaded by patients and clinicians scored 4 points, respectively (*χ*^2^ = 14.617, *p* = 0.006).

No significant differences were found in the cross-association analysis of uploader with the GQS, PEMAT-U, and PEMAT-A scores (all *p* > 0.05), indicating that different uploader groups performed comparably regarding overall quality, understandability, and actionability.

### Correlation analysis

**Figure 6:**
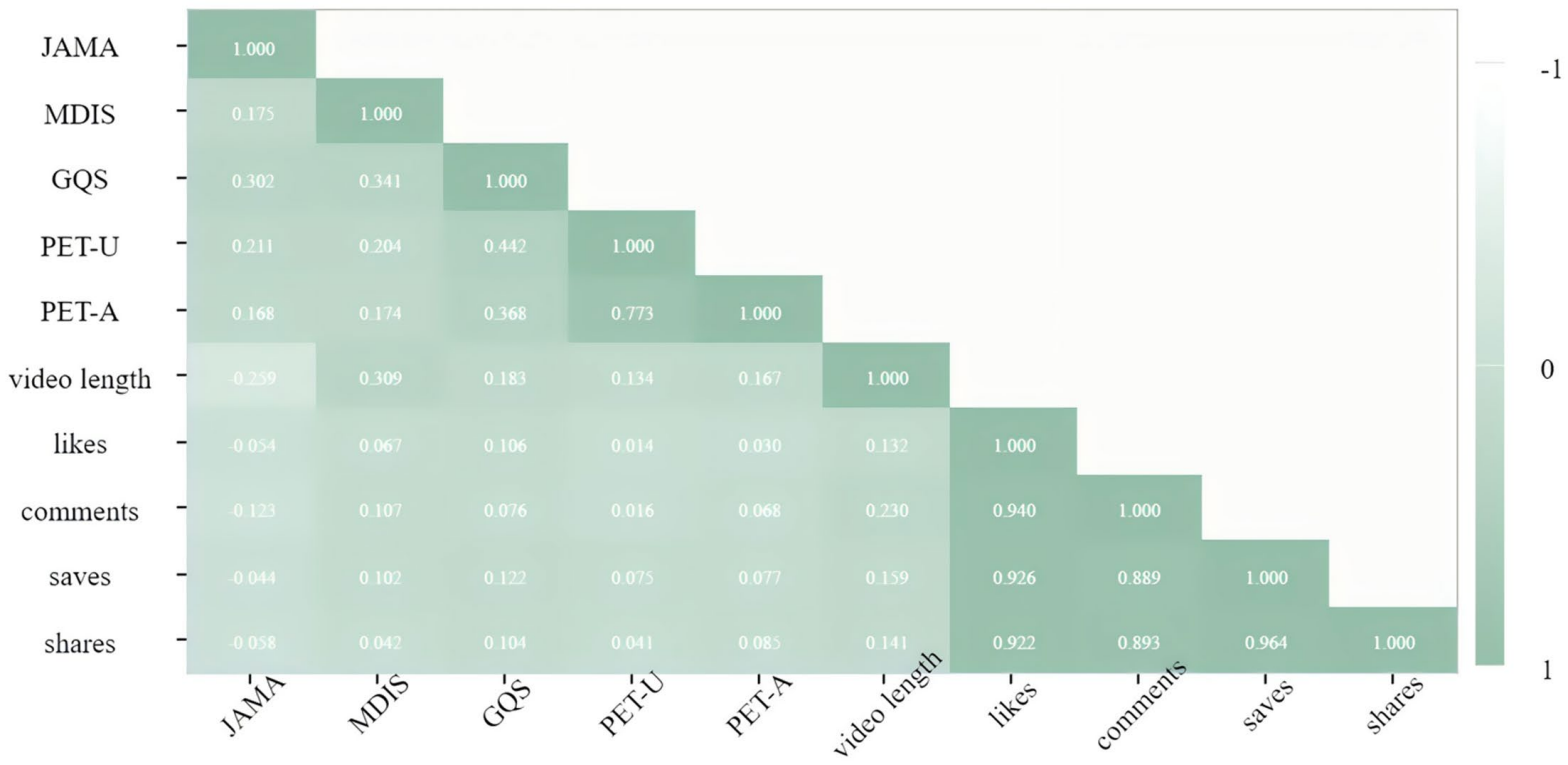
Correlation analysis between video content characteristics and quality scores. GQS, global quality scale; JAMA, *Journal of the American Medical Association*; mDISCERN, modified DISCERN; PEMAT-A, patient education materials assessment tool for audiovisual materials; PEMAT-U, patient education materials assessment tool for printable materials.

Spearman correlation analysis revealed the patterns of association between variables (Figure 6).

### Internal synergistic associations among quality metrics

The PEMAT-U and PEMAT-A scores demonstrated a strong positive correlation (*r* = 0.773, *p* < 0.001); the GQS and PEMAT-U scores showed a moderate positive correlation (*r* = 0.442, *p* < 0.001); and the JAMA and mDISCERN scores exhibited only a weak positive correlation (*r* = 0.175, *p* = 0.041).

### Bidirectional differentiation between video duration and quality

Video duration was significantly negatively correlated with the JAMA score (*r* = 0.259, *p* = 0.002); highly-significantly and positively correlated with the mDISCERN score (*r* = 0.309, *p* < 0.001); weakly-significantly and positively correlated with the GQS score (*r* = 0.183, *p* = 0.033); and not significantly correlated with the PEMAT-U score (*r* = 0.124, *p* = 0.148).

### “Complete disconnection” between interaction volume and quality

There was high internal synergy among interaction metrics, with likes and comments (r = 0.940) and favorites and shares (*r* = 0.964, both *p* < 0.001). Interaction volume showed almost no significant correlation with quality scores, with likes and the GQS score (*r* = 0.106, *p* = 0.218) and comments and the PEMAT-U score (*r* = 0.068, *p* = 0.427).

### Video content

**Figure 7:**
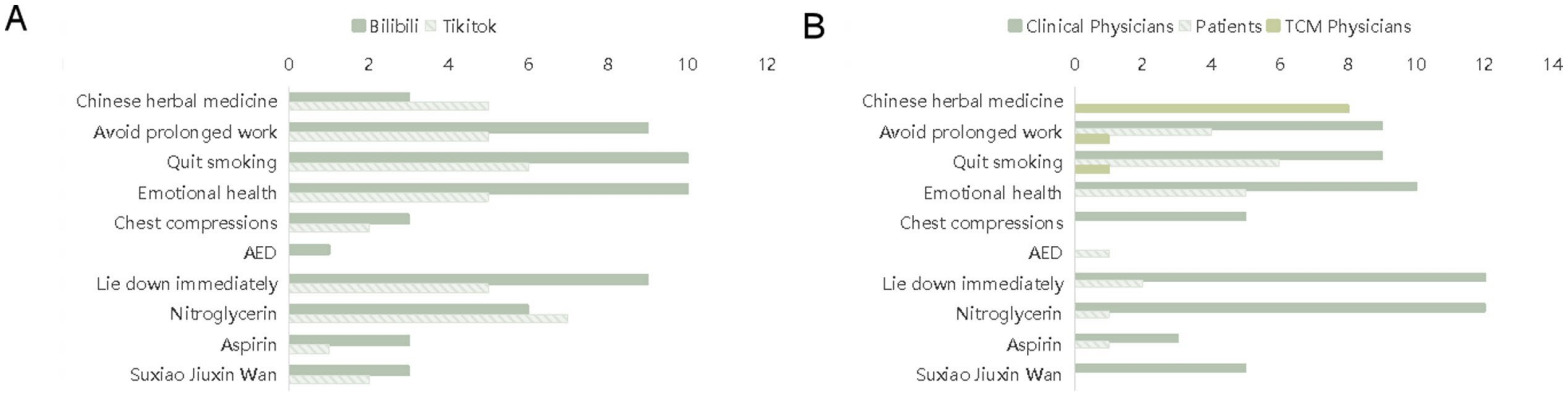
Analysis of myocardial infarction-related video content. **(A)** Distribution of various types of myocardial infarction-related content across different platforms. **(B)** Distribution of various types of myocardial infarction-related content among different uploader types. AED, automated external defibrillator; TCM, traditional Chinese medicine.

The health recommendations in the videos demonstrated an imbalanced pattern of “high coverage of basic lifestyle advice, severe deficiency in first aid and medication guidance” (Table 3 and Figure 7).

**Table 3:** Distribution of myocardial infarction-related recommended content across different platforms and uploader types (*n*, %).

	Non-pharmacological						Pharmacological			
	Lie down immediately	Avoid prolonged work	Quit smoking	Emotional healthely	Chest compressions	AED	Traditional Chinese Medicine	Nitroglycerin	Aspirin	Suxiao Jiuxin Wan
Platforms										
TikTok	9 (6.57%)	9 (6.57%)	10 (7.30%)	10 (7.30%)	3 (2.19%)	1 (0.07%)	3 (2.19%)	9 (6.57%)	6 (4.38%)	3 (2.19%)
Bilibili	5 (3.65%)	5 (3.65%)	6 (4.38%)	5 (3.65%)	2 (1.46%)	0 (0.00%)	5 (3.65%)	5 (3.65%)	7 (5.1%)	1 (0.07%)
Uploaders										
Clinical physicians	12 (8.76%)	9 (6.57%)	9 (6.57%)	10 (7.30%)	5 (3.65%)	0 (0.00%)	0 (0.00%)	12 (8.76%)	3 (2.19%)	5 (3.65%)
TCM physicians	2 (1.46%)	4 (2.92%)	6 (4.38%)	5 (3.65%)	0 (0.00%)	1 (0.07%)	8 (5.84%)	1 (0.07%)	1 (0.07%)	0 (0.00%)
Patients	0 (0.00%)	1 (0.07%)	1 (0.07%)	0 (0.00%)	0 (0.00%)	0 (0.00%)	0 (0.00%)	0 (0.00%)	0 (0.00%)	0 (0.00%)

TCM, traditional Chinese medicine; AED, automated external defibrillator.

### High popularity of basic lifestyle advice

The three types of advice—“avoid prolonged work,” “quit smoking,” and “emotional health”—were frequently mentioned on both platforms and among different uploader groups: on Bilibili, they were mentioned 9, 10, and 10 times respectively; on TikTok, 5, 6, and 5 times respectively; in clinician-uploaded videos, the mention rate of “emotional health” was > 30%.

### Significant gaps in first aid and medication advice

Regarding first aid measures, the mention rates of chest compressions and automatic external defibrillators (AED) were < 10%, with only clinician-uploaded videos mentioning them more frequently, at 5 and 12 times, respectively. Mention rates were extremely low in TCM doctor- and patient-uploaded videos on Bilibili. For medication advice, the mention rates of “nitroglycerin,” “aspirin,” and “Suxiao Jiuxin Wan” were < 5%; clinicians mentioned them three and five times; patients mentioned each of them once, and TCM doctors did not mention any of them. Moreover, some videos did not specify the applicable population or contraindications for the medications.

## Discussion

### Research background

With the rapid development of short-video platforms, social media has become an important channel for the public to access health information. Existing research has focused on analyzing MI-related videos on YouTube (11). As mainstream platforms in China, TikTok and Bilibili require critical evaluation of their health content quality. In this cross-sectional analysis of myocardial infarction–related videos on TikTok and Bilibili, we identified significant variability in quality and educational value across platforms and uploader types.

For the general public, practical information on MI warning signs, treatment, and management is more relevant than specialized content, such as the pathological mechanisms of MI. Studies demonstrate that an accurate understanding of diseases reduced patient anxiety and improve treatment adherence, which is particularly crucial for cardiovascular disease management.

### Differences between platforms

To the best of our knowledge, this study is the first to systematically compare the reliability and quality of MI-related content on two major short video platforms in China (TikTok and Bilibili), addressing the research gap in this field.

Our analysis revealed a clear distinction in content format, with Bilibili utilizing medium-to-long videos to enhance knowledge dissemination by emphasizing the completeness and depth of medical information. Conversely, TikTok was found to rely on fragmented content for efficient distribution, highlighting immediacy and transmission efficiency. This difference stems from platform positioning, with Bilibili users tending to engage in deeper viewing, whereas TikTok users are more accustomed to short browsing sessions (12).

There is a large disparity in interaction volume. The median interaction data on TikTok, including likes and comments, exceeds that on Bilibili by > 30 times in all dimensions. This pattern is closely related to the algorithmic preference on TikTok for short and fast content and also reflects user behavior differences, with TikTok users showing a stronger willingness to interact, whereas Bilibili users, despite focusing on in-depth content, have lower active interaction rates (13).

Regarding content quality and reliability assessment, a significant difference existed only in the reliability indicator (mDISCERN score), with Bilibili videos having a slightly higher proportion of high-reliability content, compared with TikTok videos. In terms of quality consistency, no significant differences were found in the credibility (JAMA score) and overall quality (GQS score) dimensions, indicating that both platforms perform comparably regarding basic information credibility, content quality control, information understandability, and actionability. Therefore, platform positioning differences may not cause systematic quality deviations, and high-quality medical information can be effectively transmitted in various formats.

### Uploader attribute differences: group characteristics influence content format while professional background influences video quality

Videos uploaded by uploaders with different professional backgrounds (patients, clinicians, and TCM doctors) demonstrated differences in video content format and quality. Patient-uploaded videos exhibited the longest median duration, followed by those uploaded by clinicians and, finally, those uploaded by TCM doctors, reflecting that patients tend to share personal illness experiences and recovery processes in detail through long videos to convey complete information. Conversely, professional physicians (both clinicians and TCM doctors) prioritize information transmission efficiency, favoring short videos that focus on core medical knowledge and eliminate redundancy.

Our study reveals a “quality-popularity paradox”: although patient-uploaded videos received the highest number of likes, they demonstrated a higher proportion of low JAMA scores, and cross-association chi-square tests showed poorer quality stability with uneven content; conversely, videos uploaded by professional doctors (e.g., clinicians), while less popular, demonstrated more stable quality and higher reliability, highlighting the conflict between platform content review mechanisms and clinical reality. Although high-traffic videos provide patients with more treatment options, they can easily lead patients to form biased perceptions due to the insufficient professional expertise of uploaders (such as overstating efficacy and ignoring adverse reactions), thereby affecting health decisions (14, 15).

In this study, videos uploaded by TCM doctors scored significantly lower on the PEMAT-U/A scales, indicating poorer understandability and actionability. We must emphasize that this finding pertains strictly to educational transferability and informational quality, and should not be misinterpreted as evidence regarding the clinical efficacy or therapeutic validity of TCM itself. The assessment instruments employed JAMA, mDISCERN, GQS, and PEMAT were all developed within the framework of evidence-based Western medicine,to assess informational quality, transparency, understandability, and actionability within a digital health communication framework. They do not evaluate therapeutic validity or clinical efficacy. Therefore, low PEMAT scores among TCM videos likely reflect a modality gap in communication design rather than a deficit in medical value. TCM explanations often involve relational concepts (e.g., qi stagnation, blood stasis, yin-yang imbalance) that resist simplification into the linear, action-oriented “bullet points” privileged by PEMAT scoring. This does not render the content clinically ineffective, but rather signals a need for improved health literacy strategies that can translate traditional medical concepts into accessible patient education without diluting their intrinsic logic.

However, introducing traditional Chinese medicine content on digital health platforms like TikTok and Bilibili presents both opportunities and challenges:

Positive aspects: TCM videos can enhance cultural resonance and accessibility for specific user groups, helping to improve overall health practice awareness and encouraging users to seek professional advice. Social media lowers the barrier to content dissemination, enabling users without access to academic or clinical literature to obtain health information.

Challenges and Risks: Without rigorous contextualization, TCM content may be misinterpreted as authoritative medical advice. Our analysis indicates that many TCM videos lack actionable details and suffer from poor structural quality, potentially hindering users’ ability to understand and implement appropriate self-management. Social media algorithms may prioritize high-engagement content over accuracy, amplifying misconceptions. These characteristics underscore the necessity for platform-level content standards, clear evidence labeling, and enhanced user health literacy.

The limitations of currently used evaluation methods reveal deeper challenges within the digital health ecosystem: the risk of cognitive bias in content assessment. As social media platforms become primary sources of health information in China, simply applying Western medical quality assessment tools to evaluate TCM content may struggle to adequately assess the traditional knowledge system. Therefore, culturally adapted assessment tools must be developed that respect the epistemological foundations of TCM theory. Taking myocardial infarction as an example, such tools could assess whether videos accurately reflect classical TCM theories, clearly communicate the scope and limitations of TCM in acute conditions like MI, and appropriately guide patients on when to seek Western medical intervention. Secondly, efforts should focus on supporting the advancement of traditional Chinese medicine practitioners. We found TCM videos to be the shortest in duration and least interactive, indicating untapped potential. By optimizing narrative design and visual aids, TCM creators can significantly enhance patient comprehension while maintaining professional integrity.

### Video quality and feature correlation analysis

A significant positive correlation was found between video understandability and actionability, indicating that videos with higher understandability can more effectively guide user behavior. Nevertheless, video duration demonstrated a two-way effect on quality: longer videos enhanced content reliability by citing medical evidence and strengthening logical chains, whereas excessive length easily led to information redundancy and deviation from the topic.

The health science popularization field shows a significant quality–popularity paradox. User interaction behaviors, including likes, comments, favorites, and shares, are highly synergistic, indicating intrinsic consistency among multi-dimensional interaction data and aligning with the findings of Liu et al. (16). However, these interaction metrics show almost no significant correlation with professional quality scores, including the JAMA, GQS, mDISCERN, and PEMAT scores. This pattern confirms that user engagement is driven mainly by content popularity rather than objective quality and reflects limitations in the ability of the public to discern the quality of health videos. This finding is not unique to China, research on Latin American TikTok medical content similarly found that engagement metrics amplify influence but fail to reliably reflect educational quality, raising global concerns about health misinformation on social media platforms (17).

Therefore, we recommend platforms should prioritize algorithms that emphasize content quality over user engagement metrics to recommend higher-quality content to users while establishing an authoritative quality labeling system to help users identify high-quality content.

### Video content analysis

Our analysis of the collected short videos revealed a significant imbalance. Basic lifestyle advice was widely covered; however, first aid and medication guidance were severely lacking. Specifically, lifestyle recommendations such as “avoid overwork,” “quit smoking,” and “emotional management” were frequently mentioned, highlighting the emphasis placed on daily health management.

Nonetheless, the popularity of professional first aid knowledge was extremely low, with “chest compressions” and “AEDs” appearing in < 10% of videos. This deficiency hinders ordinary users from acquiring key first aid skills through short videos, potentially delaying effective responses in emergency situations.

Furthermore, medication advice coverage was not only low, at < 5%, but also presented potential safety risks. Common medications, such as nitroglycerin, aspirin, and Suxiao Jiuxin Wan, were rarely discussed, and some content failed to indicate the applicable population and contraindications; for example, the risk of nitroglycerin for hypotensive patients or the contraindications of aspirin for individuals with bleeding tendencies (18, 19).

This information gap may result in user misunderstandings and even pose health risks, especially when viewers lack basic medication knowledge. Overall, this imbalance in video content not only highlights gaps in health information dissemination but may also widen the cognitive gap of the public in first aid and medication use, requiring more comprehensive content optimization to enhance educational effectiveness.

### Strengths and limitations

This study employed multiple assessment tools (JAMA benchmark criteria, mDISCERN, GQS, PEMAT-U, and PEMAT-A) to systematically evaluate the quality of MI-related videos on Bilibili and TikTok. These tools focus on different dimensions such as information coverage, publication quality, credibility, information reliability, and overall quality, achieving a multi-dimensional assessment of video quality.

Notably, our in-depth exploration examines how different platforms and uploader groups (clinicians, patients, and TCM doctors) affect video quality, as well as the correlation between video characteristics (likes, comments, favorites, shares) and quality scores. These correlation analyses significantly enhance the robustness and clinical applicability of our findings.

Nevertheless, this study has the following limitations. First, data only covered videos published before September 1, 2025, without considering the dynamic impact of platform algorithm iterations on video recommendations, which may limit the timeliness of conclusions. Second, existing tools (eg., JAMA benchmark criteria and DISCERN) are primarily designed based on modern medical standards and lack specialized assessment dimensions for TCM content, potentially leading to systematic underestimation of TCM video quality (20). Third, quality scoring relies on manual evaluation, which may introduce subjective bias (17). Fourth, the study focused solely on Chinese video platforms, and the generalizability of conclusions to videos in other languages and other cultural contexts remains to be verified (7). Thus, future research should expand the sample size to enhance statistical power, develop automated assessment tools incorporating artificial intelligence to reduce subjective bias, establish an improved assessment system including TCM dimensions, and conduct cross-language and cross-platform comparative studies.

## Conclusion

This first systematic comparison of MI-related content on TikTok and Bilibili revealed the following issues, a profound disconnect between user engagement metrics and professional quality indicators (the “quality-popularity paradox”), along with significant missing in content, particularly regarding emergency measures and medication safety information. Uploader background influences video quality, with clinicians and TCM practitioners offering higher credibility, while patients provide more detailed, relatable narratives. The low comprehensibility scores for TCM content particularly highlight gaps in our assessment tools, underscoring the need for culturally adapted evaluation frameworks.

These findings can translate into clear, actionable recommendations. Platforms must shift from algorithms prioritizing user engagement to those emphasizing content quality, implementing mandatory emergency response labels and AI-assisted safety information pre-screening. Medical institutions and professional societies should collaborate with platforms to produce standardized templates covering core information on MI emergency care and medication, using standardized, easy-to-adapt scripts and visuals. Finally, the public requires improved health literacy to bridge the disconnect between popularity and quality, enabling them to identify trustworthy information. Essentially, unlocking short-video platforms’ potential in medical information education is crucial to ensure digital health content ultimately serves population health protection (21).

## Data Availability

The original contributions presented in the study are included in the article/supplementary material, further inquiries can be directed to the corresponding author.
